# Small ruminant lentivirus infection influences expression of acute phase proteins and cathelicidin genes in milk somatic cells and peripheral blood leukocytes of dairy goats

**DOI:** 10.1186/s13567-018-0607-x

**Published:** 2018-11-13

**Authors:** Daria Reczyńska, Magdalena Zalewska, Michał Czopowicz, Jarosław Kaba, Lech Zwierzchowski, Emilia Bagnicka

**Affiliations:** 1 Department of Animal Improvement, Institute of Genetics and Animal Breeding, Polish Academy of Sciences, Postępu 38A St., 05-552 Jastrzębiec, Poland; 2 Laboratory of Veterinary Epidemiology and Economics, Faculty of Veterinary Medicine, Warsaw University of Life Sciences, Nowoursynowska 159c, 02-776 Warsaw, Poland; 3 Department of Molecular Biology, Institute of Genetics and Animal Breeding, Polish Academy of Sciences, Postępu 38A St., 05-552 Jastrzębiec, Poland

## Abstract

The aim of the study was to analyze acute phase protein and cathelicidin gene responses to small ruminant lentivirus (SRLV) infection in goats. In uninfected goats, we found higher *Cp* and lower *Fbγ* mRNA levels in blood leucocytes (BL) than in milk somatic cells (MSC), as well as lower SAA, Hp, and CRP and higher Cp and AGP concentrations in blood serum than in milk. In SRLV-infected goats, we found higher *Fbγ* and *MAP28* and lower *Cp* expression in MSC than in BL, and higher SAA, Hp, Fb, and MAP28 and lower AGP concentrations in milk than in blood serum. Higher *SAA* and *Hp* expressions in BL and *Hp* expression in MSC were found in SRLV-infected goats. In SRLV-infected goats, we observed a higher concentration of SAA in blood serum, while in milk, lower SAA, Cp, and MAP28 and higher MAP34 concentrations were observed. The expression profiles of the studied genes differed between BL/serum and MSC/milk. The elevated SAA concentration in blood serum was accompanied by a decreased concentration of SAA and Cp in the milk of infected goats. No differences in the expression of the other studied genes may mean that the SRLV has the ability to evade the immune system, continuing to replicate. The elevated concentration of SAA in blood serum may promote viral multiplication. This higher concentration of SAA in blood serum and simultaneous reduced concentration of SAA and Cp in milk may be additive indicators of this infection.

## Introduction

Small ruminant lentivirus (SRLV) causes chronic diseases in domestic and wild small ruminants. Caprine arthritis–encephalitis virus (CAEV) and visna-maedi virus (VMV), responsible for diseases in goats and sheep, respectively, were first considered to be two related but separate pathogens. However, through phylogenetic and epidemiologic analysis, they were then merged into a single species, that of SRLV. SRLV belongs to the Lentivirus genus, *Retroviridae* family together with human (HIV), simian (SIV), and feline immunodeficiency (FIV) viruses. Lentiviruses are characterized by high biodiversity—a direct consequence of their retroreplication mechanism, which results in a high number of point mutations [1]. This phenomenon suggests the possible emergence of new recombinant variants of the virus genome, which can acquire a new host (analogous to the history of SIV-HIV/AIDS) [2]. Therefore, there is a need to study all aspects of the disease, including the host’s immunological response, at the molecular level.

The main target cells for SRLV are monocytes, macrophages, and dendritic cells but not lymphocytes. SRLV cause a systemic infection that may also affect the central nervous system, in addition to the mammary gland, and respiratory and musculoskeletal system [3]. The maturation of blood monocytes into tissue macrophages initiates virus replication within these cells [4]. The disease eventually leads to emaciation and the premature culling of animals, but at different times after infection [5]. Goats with clinical symptoms of the disease suffer from arthritis, which causes severe pain. The infection is difficult to diagnose, as only 30% of the infected animals develop apparent signs [6]. Moreover, the blood antibody titer can remain below the limit of detection, even long after the infection. Thus serological ELISA (enzyme-linked immunosorbent assay), cannot be considered as a reference test [7, 8], which necessitates search for additional biomarkers of the infection. We hypothesize that the concentration or the mRNA level of some proteins involved in the immune defense against viruses could play this role.

Because human safety and animal welfare are priorities in developed societies, the eradication of SRLV has become an urgent need [2, 6]. There is currently no effective therapy against caprine arthritis–encephalitis (CAE), so the most effective way to eradicate this disease is to wean kids from infected dams immediately after birth, rearing them on bovine colostrum/milk or milk replacement, and remove infected animals from the herd [6]. Moreover, reducing the virus load in the infected animals could improve the welfare of animals and prolong their life [9, 10]. It is important to understand the complexity of pathogenesis and the precise mechanisms responsible for SRLV infection.

The molecular basis of an organism’s immune response to pathogen invasion in domestic animals has recently become one of the most intensively studied topics in the field of veterinary medicine. In this study, we focus mainly on acute phase proteins (APP) and cathelicidins. APP are various glycoproteins with diverse structures, functions, and expression levels. In general, they can be divided into two subgroups: positive APP, whose concentration increases, and negative APP, whose concentration decreases in response to the stimulus [11]. Usually, their concentrations in blood change within 24–48 h after infection, but they can also serve as an indicator of chronic inflammation [12]. In domestic animals, the positive group includes: serum amyloid A (SAA), haptoglobin (Hp), C-reactive protein (CRP), ceruloplasmin (Cp), fibrinogen (Fb), α_1_-acid glycoprotein (AGP), and lactoferrin (Lf). The negative group includes albumins, transferrin (Tf), and transthyretin (TTR) [13]. Thus far, only five proteins have been identified in goats as positive APP—SAA, Hp, Cp, Fb, AGP and one negative—alpha-lactalbumin (LALBA). Their expression has been detected in a broad range of cells, such as hepatocytes, and leukocytes or epithelial cells of various organs [14].

Cathelicidins are an another important component of the innate immune system. They are small cationic peptides belonging to a wide, heterogeneous group of cationic antimicrobial peptides (AMP). They exhibit high activity not only against bacteria but also against enveloped viruses such as vaccinia virus, respiratory syncytial virus, influenza A virus, HIV, herpes simplex virus, and dengue virus [15]. In goats, five cathelicidins have been described so far: *Capra hirca* bactenecin 3.4 (ChBAC3.4), bactenecin 5 (BAC5), bactenecin 7.5 (BAC7.5), cathelicidin 6 (MAP28), and cathelicidin 7 (MAP34). They show activity against Gram-negative and Gram-positive bacteria, as well as fungi and enveloped viruses [16–18].

As mentioned, monocytes, macrophages, and mammary epithelial cells are the target cells for SRLV. Moreover, epithelial cells of the mammary gland can act as viral reservoirs [19]. Since milk somatic cells (MSC), in addition to macrophages and neutrophils, also consist of live, exfoliated mammary epithelial cells [20], it is possible to study changes occurring not only in leukocytes but also in the SRLV-infected mammary epithelium, using a non-invasive method [21]. The aim of this study was to analyze the response of APP and cathelicidin genes to SRLV infection. Blood leukocytes (BL) and MSC were used in the present study to determine the mRNA levels of selected genes participating in the immune response to the viral infection (*SAA*, *Hp*, *CRP*, *Cp*, *Fbα, Fbβ*, *Fbγ*, *AGP*, *LALBA*, *ChBAC3.4*, *BAC5*, *BAC7.5*, *MAP28*, and *MAP34*). In addition, ELISA was used to measure the concentrations of SAA, Hp, CRP, Cp, Fb, AGP, LALBA, MAP28, and MAP34 proteins in the blood serum and milk of uninfected and asymptomatically infected goats.

## Materials and methods

### Animals

Twenty-four dairy goats of the Polish White Improved (PWI) and Polish Fawn Improved (PFI) breeds were used in the study. They were kept in a loose barn with free access to water and a salt lick. The goats were fed according to a system developed by the Institut National de la Recherche Agronomique (INRA) of France and adopted by the National Research Institute of Animal Production (IZ PIB-INRA), Poland [22]. The basic diet consisted of maize silage, wilted grass silage, and concentrates. Goats were machine-milked twice a day. Animals were kept under the constant care of a veterinarian. For the preceding 20 years, goats from this herd had been tested serologically for SRLV twice a year (in November and June) as a part of another long-term research project.

Goats were divided into two groups: Group 1 goats were free from SRLV infection (*N* = 12) and Group 2 goats were naturally infected with SRLV (*N* = 12). Confirmation of infection was based on at least two consecutive positive serological results. Irrespectively of the serological status, all goats under study were asymptomatic. During the study, tests were also performed twice a year to identify new potential infections and eliminate infected animals from the control group. All serological examinations were performed using ELISA (ID Screen^®^ MVV-CAEV, Indirect Screening ELISA, IDvet Innovative Diagnostics, Grabels, France) [23]. All goats, both healthy and infected, were held in the same environmental conditions, but separately. Goats free from SRLV were milked first to eliminate the risk of SRLV transfer via milking equipment.

Both groups were identical in terms of breed and parity. In each group, six goats of both breeds (PWI and PFI) were allocated. For each breed, there were three goats in the 2nd and three in the 4th or further lactation.

To include the changes occurring in the organisms of dairy goats in the statistical analysis, milk and blood samples were collected five times during the morning milkings during two lactations: just after kidding and on days 30 (early lactation), 60 (peak lactation), 140 (mid-lactation), and 200 (late lactation). To obtain MSC for RNA isolation, 1 L of milk was collected into a plastic, RNase-free bottle. Just before the milk sampling, a small amount of foremilk was also collected by hand in a sterile manner. Microbiological tests of the milk were conducted to exclude the interference of pathogenic microorganisms other than SRLV. On the same day, whole blood samples were collected by a veterinarian into S-Monovette^®^ 9 mL EDTA tubes as well as into 9 mL tubes with clot-activator (Sarstedt AG & Co., Germany). Blood was centrifuged at 3000 rpm for 20 min at 4 °C. The serum was harvested and frozen at −80 °C for further analysis.

### Microbiological analysis of milk

To identify the bacterial pathogens, Columbia agar supplemented with 5% sheep blood and MacConkey agar (bioMérieux, France) were used. Both media were inoculated with 100 μL of milk. Plates were incubated at 37 °C for 48 h. To identify the bacteria species, VITEK 2 equipment was used (bioMérieux).

### RNA isolation from MSC and BL

One liter of milk was centrifuged at 1500 rpm for 30 min, and skimmed milk and fat phase were removed. The pellet of somatic cells was transferred to 50 mL Falcon™ Conical Centrifuge Tubes (Thermo Fisher Scientific, Poland) and washed with phosphate buffered saline (PBS); tubes were then centrifuged at 1100 rpm for 15 min. This operation was performed twice. The somatic cells were suspended in 1 mL TRIzol reagent (Invitrogen, USA) and stored at −80 °C for further analysis. RNA was isolated using a PureLink RNA Mini Kit (Ambion, USA) according to the manufacturer’s protocol.

Total RNA from whole blood was isolated using RNA Blood Kit (Macherey–Nagel, Germany) according to the manufacturer’s protocol.

Qualitative and quantitative analyses of RNA were performed using a NanoDrop 2000 spectrophotometer (NanoDrop, USA) and a 2100 BioAnalyzer (Agilent Technologies, France). Samples with RNA integrity number (RIN) values of more than seven were selected. Reverse transcription reactions were performed using the Transcriptor First Strand cDNA Synthesis Kit (Roche, Switzerland) according to the manufacturer’s protocol. The cDNA samples were diluted to 50 ng/µL on a 386-well plate and stored at −20 °C for further analysis.

### Real-time PCR

The expression of APP (*SAA*, *Hp*, *CRP*, *Cp*, *Fbα*, *Fbβ*, *Fbγ*, *AGP*, *LALBA*) and cathelicidins (*ChBAC3.4*, *BAC5*, *BAC7.5*, *MAP28*, and *MAP34*) genes was measured. Real-time PCR was performed using the LightCycler 480 System (Roche, Switzerland). The sequences of primers used in the analysis and the sizes of amplification products are shown in Table 1. To each sample, 2 µL of the diluted cDNA was added, as well as 11 µL of a mixture containing: 3 µL of water, 6.6 µL of SYBR Green Master Mix (Roche Diagnostics), 0.7 µL of primer F (forward), and 0.7 µL of primer R (reverse). For all genes, the same LightCycler 480 software program was set, with pre-incubation at 95 °C for 5 min (one cycle) and melting curve at 95 °C for 5 s and at 65 °C for 1 min (one cycle). The negative control was also included. Serial twofold dilutions of template cDNA (1, 1/2, 1/4, 1/16, 1/64) were also performed to determine reaction efficacy and plot calibration curve. Information on the primer annealing temperatures and cycle numbers is also shown in Table 1. The presence of the product of interest was confirmed by electrophoresis in 2% agarose gel (visualization of product size with UV light by G-BOX device (Syngene, UK)). The cyclophilin A gene (*PPIA*) was used as a Ref. [24].

**Table 1:** **Primer sequences, position in gene, accession number in GenBank, and product sizes used in qPCR**

Gene name	Gene symbol	Primer sequence		Product size (bp)	GenBank/UniProt assession^a^	References
Serum amyloid A3 protein	*SAA*	F	CTGGGCTGCTAAAGTGATCAGTAAC	69	EU884570.1	[59]
		R	CCCTTGAGCAGAGGGTCTGTGATT			
Haptoglobin	*Hp*	F	TAATGCCCATCTGCCTAC	162	XM_005692202.3	[60]
		R	CGCCCTCATAGTGTTTCA			
C-reactive protein	*CRP*	F	CTGGCTTGGGAGATTG	134	XM_018046353.1	[60]
		R	AGTGAGGGTAAGGGATT			
α-lactalbumin	*LLBA*	F	TGACATTTGTGTGTGCCAAGA	198	NM_001285635.1	PRIMER3^b^
		R	CAAGGGGGTACAAAGAAGCA			
α-acid glycoprotein	*AGP*	F	TTGCTTGGCTGCAGGTGT	197	XM_012152252.2	[11]
		R	CAATGGTCTGGTACTCTCTCTG			
Ceruloplasmin	*Cp*	F	GAGCATGAAGGGGCCATTTATC	130	NM_001256556.1	[61]
		R	GCTGTCTTCCTCACCAGG			
Fibrinogen α chain	*Fbα*	F	TGAGATCCTGAGGCGCAAAG	104	NM_001033626.1	[61]
		R	TGTCCACCTCCAATCGTTTCAT			
Fibrinogen β chain	*Fbβ*	F	GACAACGACGGCTGGAAAAC	124	NM_001142917	[61]
		R	ACGCTCCACCCCAGTAGTAT			
Fibrinogen ɣ	*Fbɣ*	F	TGCCAATAAGGGGGCCAAAG	134	NM_173911	[61]
		R	GACTGCCATCAAGCCTCTTCT			
Bactenecin-5; cathelicidin-2	*BAC5*	F	GTGGAATTCACGGTGAAGGAGAC	390	Y18873.1	[52]
		R	CTCAGGCCAAATGAGAT			
Bactenecin-7.5; cathelicidin-3	*BAC7.5*	F	GTGGAATTCACGGTGAAGGAGAC	383	AJ243125.1	[52]
		R	AGTGCTAACCTTGATGTT			
Cathelicidin-6	*MAP28*	F	GTGGAATTCACGGTGAAGGAGAG	225	AJ243126.1	[52]
		R	AATTGGGCCGACTTTGTGCC			
Cathelicidin-7	*MAP34*	F	ACCGAATTCAGCTACAGGGAGGCCGT	428	AJ243127.1	[52]
		R	ACCTGATCCTTAGGACTTC			

bp: base pairs, F: forward primer, R: reverse primer.

^a^NCBI [62].

^b^PRIMER3 [63].

### Elisa

APP and cathelicidins in plasma and milk were detected by ELISA and measured using a Sunrise™ microplate reader and the Magellan™ program (both from Tecan, Switzerland). Quantitation of selected proteins was carried out according to the manufacturer’s protocols (SunRed, China—Cp, Fb, AGP, MAP28, MAP34; Fine Test, China—SAA, Hp, CRP, LALBA, MAP28). ELISA kits for BAC5 and BAC7.5 were not commercially available. Data were obtained on the basis of average duplicate readings for each standard, control, and sample at 450 nm. A standard curve was generated, and a four parameter logistic curve-fit was done to calculate the results. The concentration of the tested protein is shown in ng/mL.

### Statistical analysis

The C_T_ values were calculated according to Pfaffl’s [25] mathematical formula:

$${\text{ratio}}\,{\text{ = }}\,\frac{{\left( {E_{{{\text{target}}}} } \right)^{{\Delta {\text{CP}}_{{{\text{target}}}} (control\, - \,sample)}} }}{{\left( {E_{{{\text{ref}}}} } \right)^{{\Delta {\text{CP}}_{{{\text{ref}}}} (control\, - \,sample)}} }}$$

where: ratio—expressed in a sample versus a control in comparison to a reference gene, ∆CP_target_—CP deviation of control-sample of the target gene transcript, ∆CP_ref_—CP deviation of control-sample gene reference transcript, E—real-time PCR efficiency of target (E(target)) or reference (E(ref)) gene transcript.

To search for differences in gene expression levels between groups of animals and types of biological material, analyses of variance were performed using the GLM procedure with Tukey–Kramer tests (SAS/STAT), taking into account the fixed effect of the interaction of the biological material (blood or milk) and the health state of the animals (SRLV-infected or uninfected), the fixed effect of lactation stage, and error as random:

$$y_{ijkl} = \mu\,+ \left( {SRLV \times TM} \right)_{ij} + SL_{k} + e_{ijkl}$$

where: y_ijkl_—trait value (gene expression), μ—overall mean, (SRLV × TM)_ij_—fixed effect of the interaction of the type of biological material (blood or milk) and the health state of the animals (SRLV-infected or uninfected) (i*j = 1, … 4), SL_k_—fixed effect of j-th stage of lactation (k = 1,…5), e_ijkl_—random error.

The normality of the distribution of all traits was checked using a Univariate procedure (SAS/STAT), and values for the expression of genes at the mRNA level were transformed into a natural logarithmic scale.

The final model did not contain the year of sampling, breed, lactation number, or lack/presence of bacteria because in prior preliminary analyses, these parameters were not found to impact the results.

## Results

All milk samples were free from bacterial pathogens; however, in 57% of the samples, environmental bacteria such as *Staphylococcus caprae*, *Staphylococcus xylosus*, and *Staphylococcus lentus* were identified (in both groups of animals and at similar levels). As mentioned above, preliminary analysis revealed that these bacteria did not influence the expression of the genes studied. Therefore, their effect was not discussed in the paper.

In this study, transcripts of *SAA*, *Hp*, *Cp*, *Fbα, Fbγ*, *AGP4*, *LALBA*, *CRP*, *BAC5*, *BAC7.5*, *MAP28*, and *MAP34* genes were found in all BL and MSC samples of both SRLV-infected and uninfected goats; however, the levels of some of these differed between the groups and types of biological materials.

**Figure 1:**
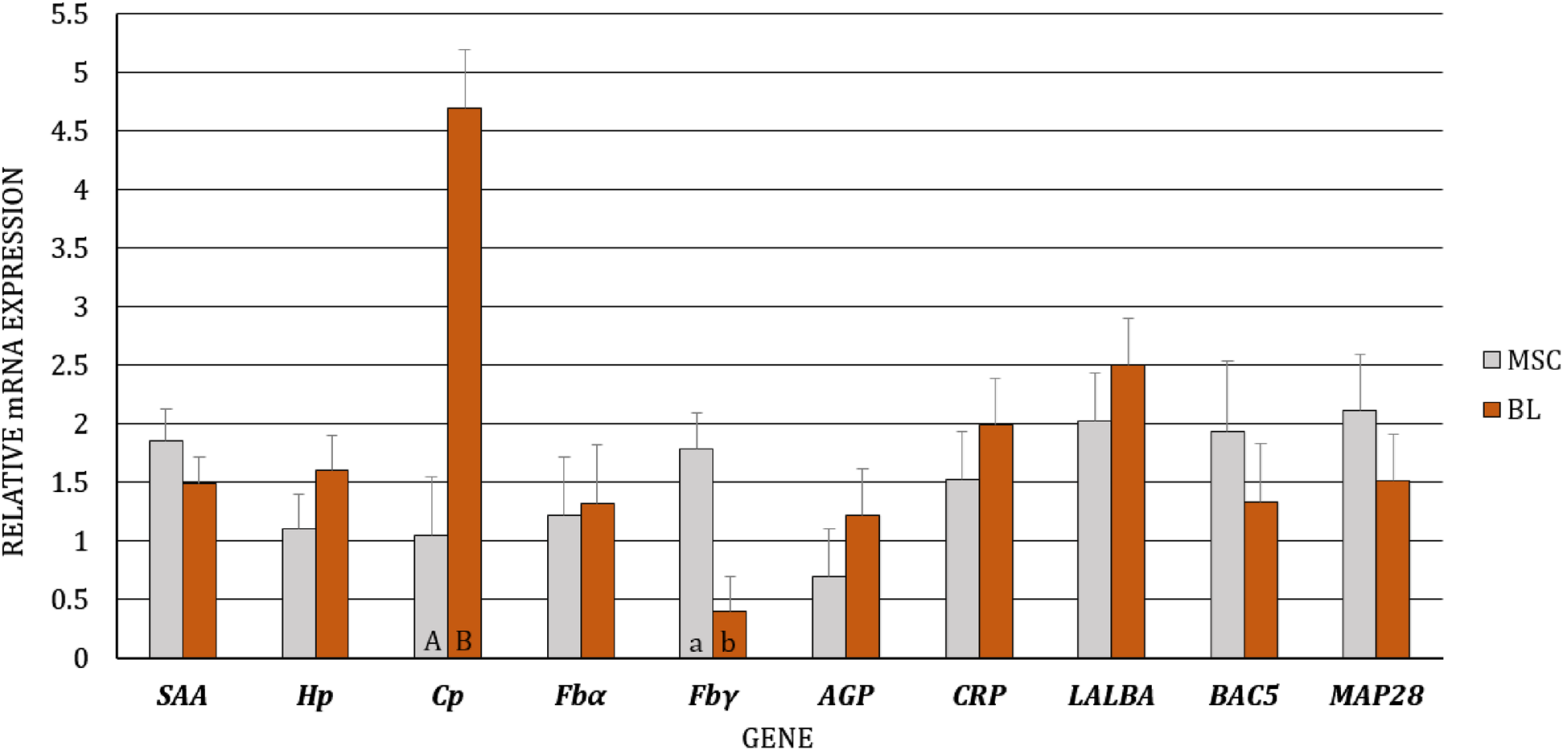
**Least-squares means (LSMEANS) and their standard errors (SE) of the mRNA levels in milk somatic cells (MSC) and blood leukocytes (BL) of goats free from small ruminant lentivirus (SRLV) infection.** SAA, serum amyloid A gene; Hp, haptoglobin gene; Cp, ceruloplasmin gene; Fbα, fibrinogen alpha gene; Fbγ, fibrinogen gamma gene; AGP, α1-acid glycoprotein gene; CRP, C-reactive protein gene; LALBA, alpha-lactalbumin gene; BAC5, bactenecin 5 gene; MAP28, cathelicidin 6 gene. The value within the same gene with different letters differs significantly between MSC and BL: a, b—at *p* ≤ 0.05 and A, B—at *p* ≤ 0.01.

**Figure 2:**
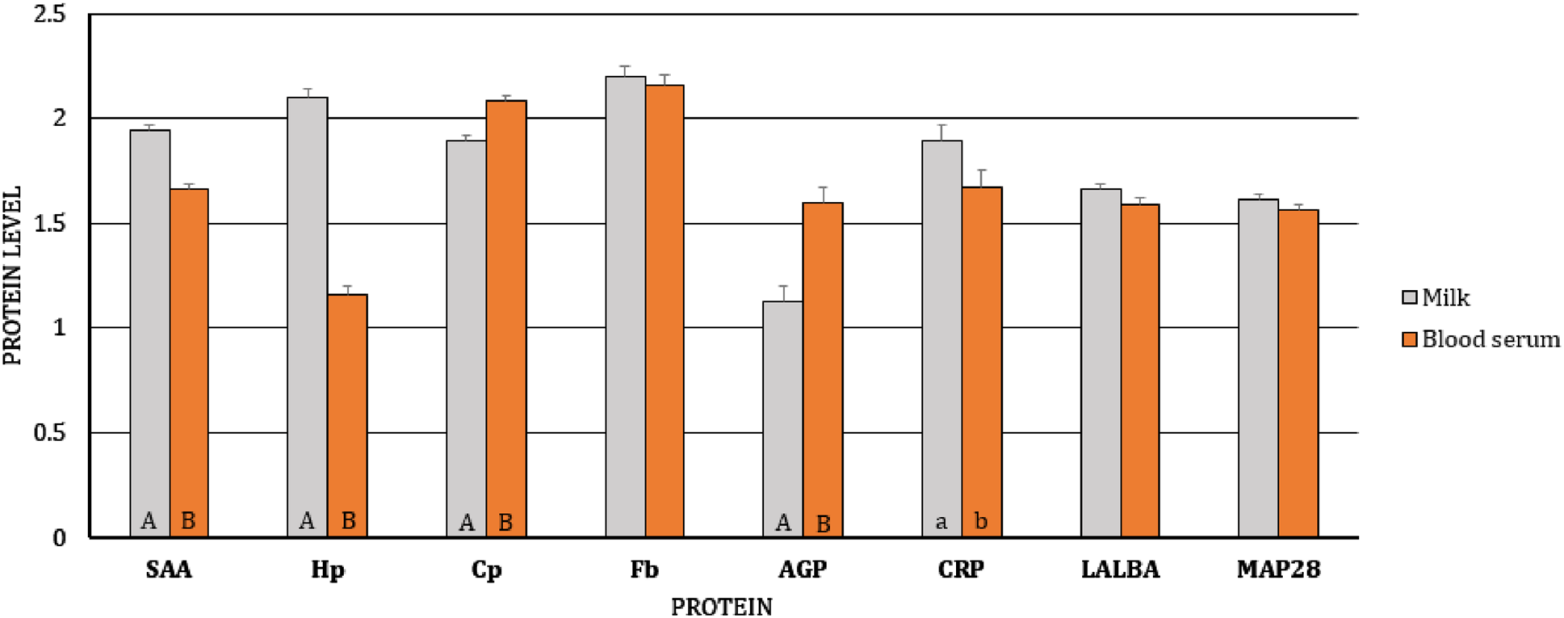
**Least-squares means (LSMEANS) (ng/mL) and their standard errors (SE) of protein levels in milk and blood serum of goats free from small ruminant lentivirus (SRLV) infection.** SAA, serum amyloid A; Hp, haptoglobin; Cp, ceruloplasmin; Fb, fibrinogen; AGP, α1-acid glycoprotein; CRP, C-reactive protein; LALBA, alpha-lactalbumin; MAP28, cathelicidin 6. The value within the same gene with different letters differs significantly between milk and blood serum: a, b—at *p* ≤ 0.05 and A, B—at *p* ≤ 0.01.

**Figure 3:**
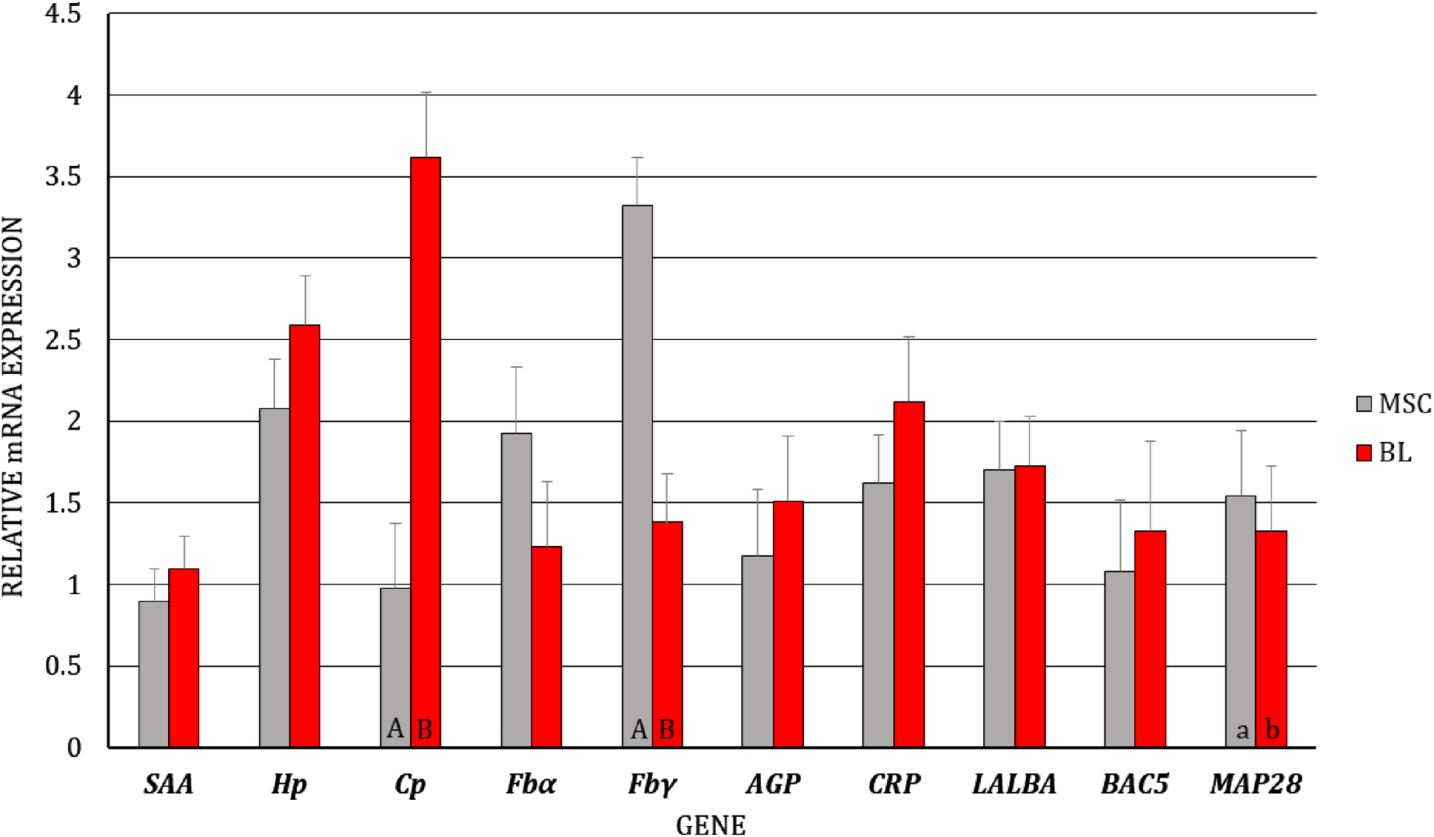
**Least-squares means (LSMEANS) and their standard errors (SE) of the mRNA levels in milk somatic cells (MSC) and blood leukocytes (BL) of goats infected with small ruminant lentivirus (SRLV).** SAA, serum amyloid A gene; Hp, haptoglobin gene; Cp, ceruloplasmin gene; Fbα, fibrinogen alpha gene; Fbγ, fibrinogen gamma gene; AGP, α1-acid glycoprotein gene; CRP, C-reactive protein gene; LALBA, alpha-lactalbumin gene; BAC5, bactenecin 5 gene; MAP28, cathelicidin 6 gene. The value within the same gene with different letters differs significantly between MSC and BL: a, b—at *p* ≤ 0.05 and A, B—at *p* ≤ 0.01.

**Figure 4:**
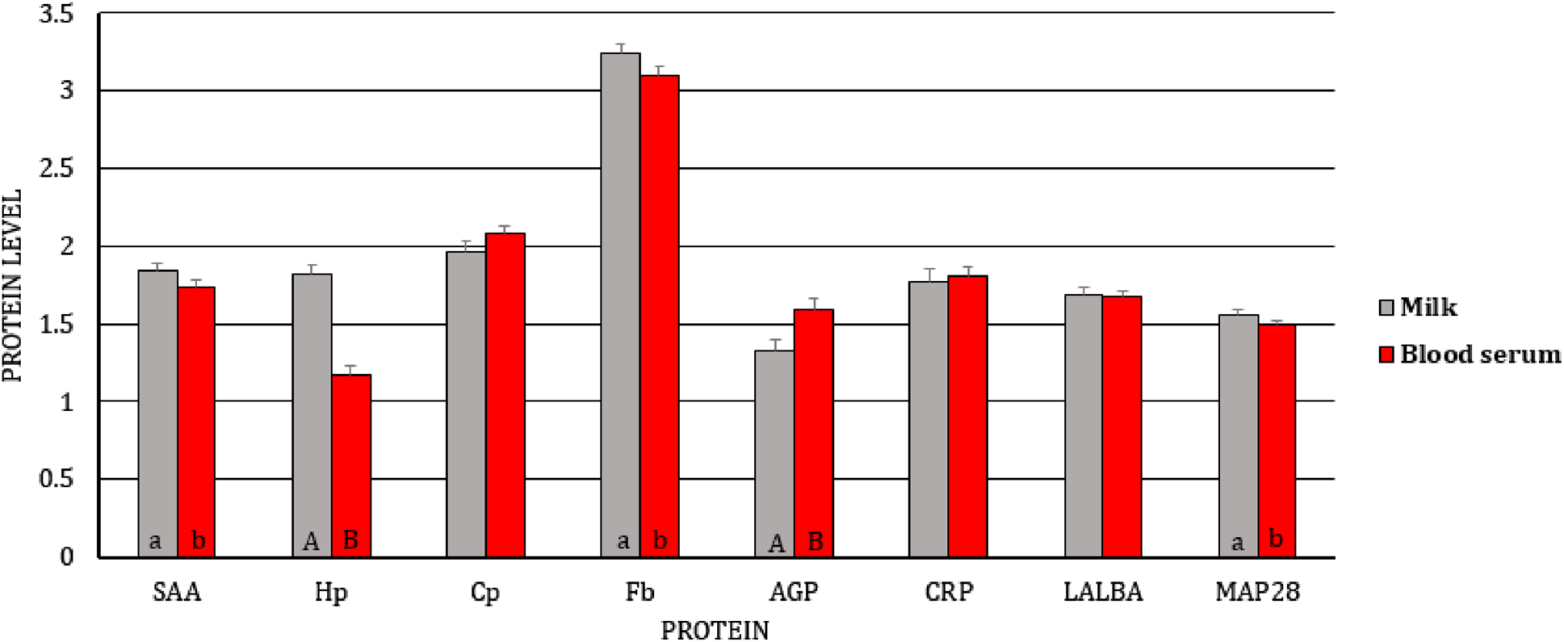
**Least-squares means (LSMEANS) (ng/mL) and their standard errors (SE) of protein levels in milk and blood serum of goats infected with small ruminant lentivirus (SRLV).** SAA, serum amyloid A; Hp, haptoglobin; Cp, ceruloplasmin; Fb, fibrinogen; AGP, α1-acid glycoprotein; CRP, C-reactive protein; LALBA, alpha-lactalbumin; MAP28, cathelicidin 6. The value within the same gene with different letters differs significantly between milk and blood serum: a, b—at *p* ≤ 0.05 and A, B—at *p* ≤ 0.01.

First, we analyzed the differences in gene expression levels between BL/blood serum and MSC/milk of uninfected and infected goats, separately (i.e., the gene expression levels of BL/blood serum were compared to the expression levels of MSC/milk in uninfected goats, and the same analysis was performed for infected goats). We observed dissimilarity in the transcript levels of *Cp* (*p* < 0.01) and *Fbγ* (*p* < 0.05) between MSC and BL of the uninfected goats, with higher expression of *Cp* and lower expression of *Fbγ* in BL (Figure 1). At the protein level, differences were found for SAA, Hp, Cp, AGP, and CRP, with lower expression of SAA (*p* ≤ 0.01), Hp (*p* ≤ 0.01), and CRP (*p* ≤ 0.05) and higher expression of Cp (*p* ≤ 0.01) and AGP (*p* ≤ 0.01) in the blood serum than in the milk of uninfected goats (Figure 2). In SRLV-infected goats, at the mRNA level, in addition to *Cp* (*p* ≤ 0.01) and *Fbγ* (*p* ≤ 0.01), expression of the *MAP28* gene was also found to be higher in MSC than in BL (*p* ≤ 0.05) (Figure 3). At the protein level, only SAA, Hp, and AGP had the same pattern of changes both in SRLV-infected and uninfected goats. In infected animals, there were no differences in Cp or CRP concentrations; however, in addition to SAA, Hp, and AGP (the same pattern as in uninfected goats), differences were found for Fb and MAP28 with their lower levels in blood serum than in milk (*p* ≤ 0.05) (Figure 4).

**Figure 5:**
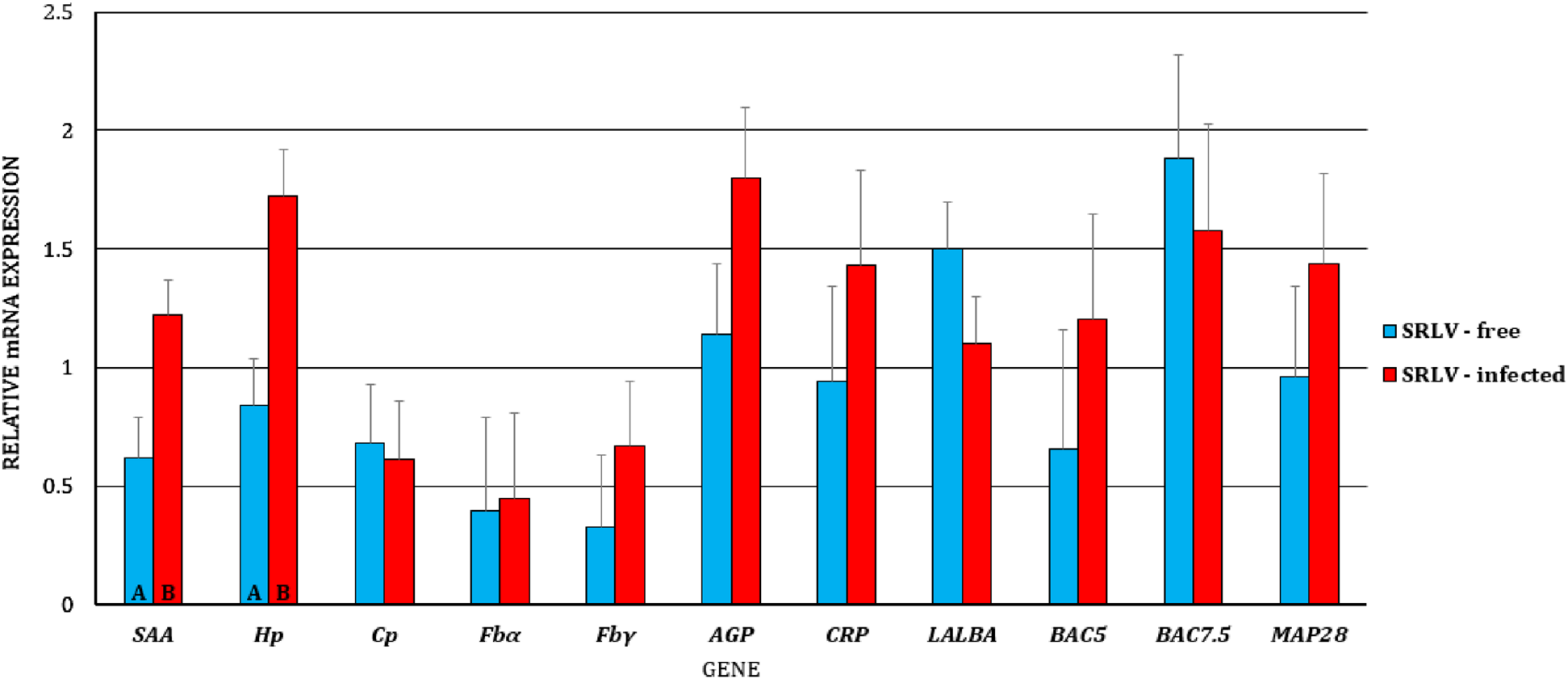
**Least-squares means (LSMEANS) and their standard errors (SE) of mRNA levels in blood leukocytes (BL) of small ruminant lentivirus (SRLV)-infected and uninfected goats.** SAA, serum amyloid A gene; Hp, haptoglobin gene; Cp, ceruloplasmin gene; Fbα, fibrinogen alpha gene; Fbγ, fibrinogen gamma gene; AGP, α1-acid glycoprotein gene; CRP, C-reactive protein gene; LALBA, alpha-lactalbumin gene; BAC5, bactenecin 5 gene; BAC7.5, bactenecin 7.5 gene; MAP28, cathelicidin 6 gene. The value within the same gene with different letters differs significantly between SRLV-infected and uninfected goats: A, B—at *p* ≤ 0.01.

**Figure 6:**
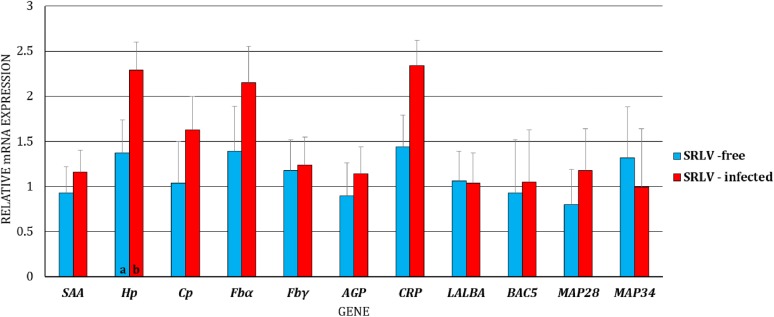
**Least-squares means (LSMEANS) and their standard errors (SE) of mRNA level in milk somatic cells (MSC) of small ruminant lentivirus (SRLV)-infected and uninfected goats.** SAA, serum amyloid A gene; Hp, haptoglobin gene; Cp, ceruloplasmin gene; Fbα, fibrinogen alpha gene; Fbγ, fibrinogen gamma gene; AGP, α1-acid glycoprotein gene; CRP, C-reactive protein gene; LALBA, alpha-lactalbumin gene; BAC5, bactenecin 5 gene; MAP28, cathelicidin 6 gene; MAP34, cathelicidin 7 gene. The value within the same gene with different letters differs significantly between SRLV-infected and uninfected goats: a, b—at *p ≤ *0.05.

The second part of the analysis concerned the comparison of mRNA and protein levels in BL or blood serum and MSC or milk between SRLV-infected and uninfected goats. We found higher expression of *SAA* and *Hp* genes (*p* ≤ 0.01) in BL of SRLV-infected goats (Figure 5). There were no significant differences between SRLV-infected and uninfected goats in the expression patterns of the other studied APP in BL. Moreover, in BL, no changes in the expression profile of cathelicidins were detected (Figure 5). Only elevated expression of the *Hp* gene was found (*p* ≤ 0.05) in MSC from SRLV-infected goats (Figure 6). No differences in *SAA* gene expression level in MSC were observed.

**Figure 7:**
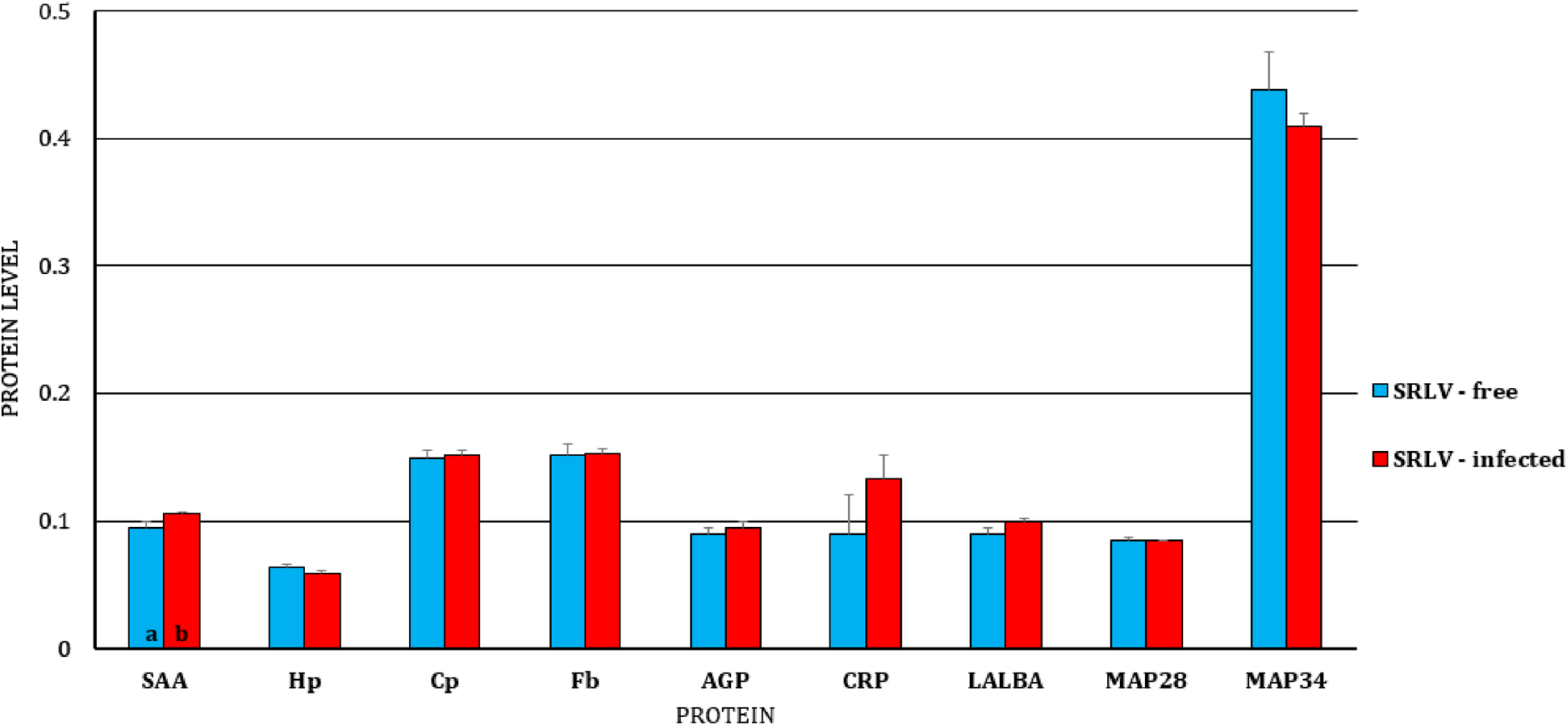
**Least-squares means (LSMEANS) (ng/mL) and their standard errors (SE) of protein level in blood serum of small ruminant lentivirus (SRLV)-infected and uninfected goats.** SAA, serum amyloid A; Hp, haptoglobin; Cp, ceruloplasmin; Fb, fibrinogen; AGP, α1-acid glycoprotein; CRP, C-reactive protein; LALBA, alpha-lactalbumin; MAP28, cathelicidin 6; MAP34, cathelicidin 7. The value within the same gene with different letters differs significantly between SRLV-infected and uninfected goats: a, b—at *p* ≤ 0.05.

**Figure 8:**
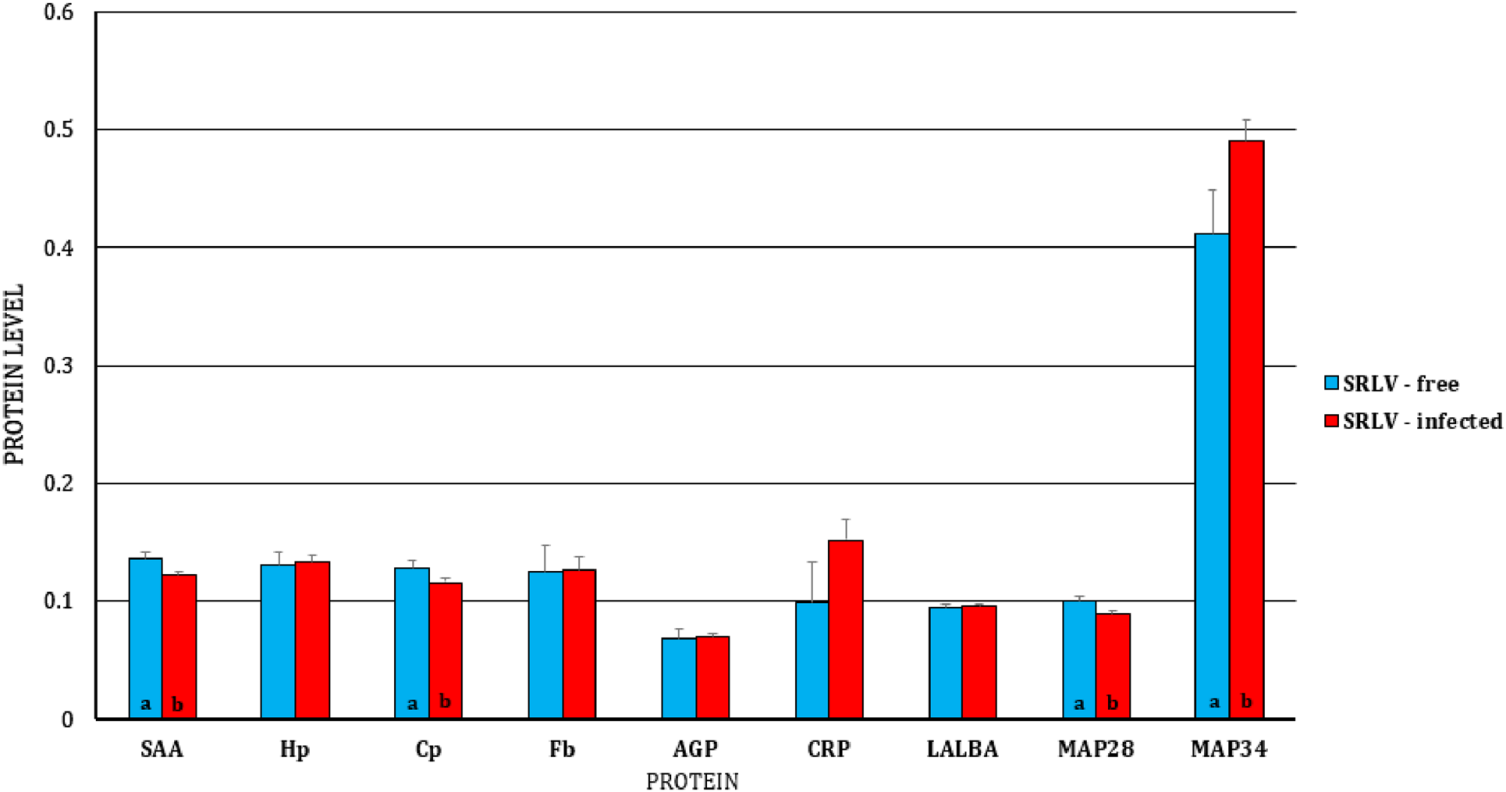
**Least-squares means (LSMEANS) (ng/mL) and their standard errors (SE) of protein levels in the milk of small ruminant lentivirus (SRLV)-infected and uninfected goats.** SAA, serum amyloid A; Hp, haptoglobin; Cp, ceruloplasmin; Fb, fibrinogen; AGP, α1-acid glycoprotein; CRP, C-reactive protein; LALBA, alpha-lactalbumin; MAP28, cathelicidin 6; MAP34, cathelicidin 7. The value within the same gene with different letters differs significantly between SRLV-infected and uninfected goats: a, b—at *p* ≤ 0.05.

Regarding the expression of the studied genes at the protein level, in BL, only a higher expression of SAA in SRLV-infected animals was found (Figure 7), while in MSC, lower expressions of SAA, Cp, and MAP28 and a higher expression of MAP34 in infected animals was observed (Figure 8).

## Discussion

APP are well-known elements of the immune system that react to various types of infection. Proteins from this group participate in the response to infections with different species of lentiviruses. As one of the activities of Cp and Fb is the removal of reactive oxygen species, their increased concentration may indicate their role in anti-oxidative processes in serum or milk. SAA, showing chemotactic activity toward leukocytes, plays an essential role in the pathogenesis of chronic inflammation, and it can also serve as an early marker of inflammation. On the contrary, this APP may inhibit antibody production [26]. Moreover, SAA seems to have an ability to stimulate the differentiation of monocytes to macrophages, which is essential for viral multiplication [27]. Hp, responsible for the uptake of free hemoglobin from blood, prevents oxidative activity and therefore has an anti-oxidative and bacteriostatic effect against iron-utilizing bacteria [12, 28, 29]. Hp is produced not only by hepatocytes but also in some mammary gland tissues (e.g., the parenchyma, the tissues surrounding the cisternal milk ducts, and in the teat). Its concentration is increased both in milk and blood during inflammation of the mammary gland in dairy cows [30]. AGP was also produced in the mammary gland ductal and cisternal tissues (elevated mRNA level), as well as in alveoli (decreased mRNA level) during experimental infection with *S. aureus* in dairy cows [31]. AGP acts as an immunomodulatory and anti-inflammatory factor, and it protects tissues from excessive damage caused by the inflammation. However, in one study in cattle, it was also shown to inhibit neutrophil migration to the site of infection and thus to elevate mortality risk of the animal [28].

We found differences in the expression of *Cp* and *Fbγ* genes, as well as the concentrations of SAA, Hp, and AGP proteins between MSC/milk and BL/blood serum of both infected and uninfected animals (this analysis was conducted separately for infected and uninfected animals). The expression of genes encoding APP showed a tissue-specific pattern that differed slightly between infected and uninfected animals. Thus, different APP genes may play an important role in the maintenance of homeostasis in various tissues/organs. In both groups of animals—infected and uninfected—the expression of different genes was up- or down-regulated at the mRNA and protein levels. Our findings are in accordance with the results of other authors who have shown various concentrations of APP in different tissues. Ametaj et al. [28] summarized that SAA3 was produced by adipose tissue, macrophages, and epithelial cells of the intestine and mammary gland. Molenaar et al. [32] found expression of SAA3 in the epithelial cells lining the ductal tissue of the teat, but not in alveoli, during lactation. However, many others have shown increased expression of SAA3 in cow and ewe milk during mastitis [33–35].

Changes in concentrations of APP have been observed in both infectious (bacterial and viral) [36] and non-infectious [37, 38] diseases. It has also been shown that the levels of SAA and Hp increase during lentiviral infection in cats [1, 39]. Moreover, upon lipopolysaccharide (LPS) infusion, elevated levels of Hp and SAA in goat milk and in the blood of goats with sub-acute ruminal acidosis were also found [11, 38, 40]. Hp and SAA have been identified as the main APP in goats; however, after injection of turpentine oil resulting in tissue irritation and an inflammatory reaction, other APP, such as Fb, were also found to increase, while albumin decreased [14].

In the second part of the study, we found higher expressions of *SAA* and *Hp* mRNAs in BL and an elevated concentration of SAA proteins in the blood serum of SRLV-infected goats. Although *Hp* gene mRNA was increased in MSC of SRLV-infected goats, there were no differences in Hp concentrations in milk. On the contrary, the concentrations of SAA and CP were lower in the milk of infected animals. However, in our previous study [7], we found higher concentrations of both SAA and Hp only in goats with apparent CAE, while the concentrations did not differ between asymptomatically infected and uninfected goats. We concluded from those results that the concentration of both APP may have increased in response to local joint inflammation, rather than to any general inflammatory processes caused by SRLV infection. However, there is a discrepancy between the results presented here and those obtained in our previous study [7] because here, we show a significant difference in SAA level in BL between uninfected and asymptomatic, infected goats.

The lack of difference in concentration of APP (except SAA, which probably assists in viral multiplication) in blood serum between uninfected and infected goats may mean that the virus is able to inhibit humoral immune response. Thus, the virus might be able to suppress the host defense against infection or to modify the immune response. This assumption was consistent with the results obtained by Crespo et al. [41]. Similar conclusions have also been presented by other authors, who stress that lentiviruses have the ability of immune evasion, continuing to replicate and gradually increasing their pathogenicity [42–44]. As stressed by Larusskain and Jugo [45] the SRLV could be invisible to the immune system of the host. Moreover, as suggested by Murphy [46], some lentiviruses can produce chemokine mimics that can inhibit host chemokines and help viruses infect target cells or disseminate in the host. Moreover, Pyrah and Watt [47] found that SRLV-infected sheep had a lower immune response to mycobacterial antigens, suggesting that the immunity of the host could be affected. SRLV’s ability to inhibit the inflammatory reaction may be the reason that infected animals are asymptomatic for months or even years after the initial infection. Furthermore, the antibody titer fluctuates, for example, during pregnancy, and drops below the limit of detection even in animals with clinical signs of the disease [48–50].

Cathelicidins are also species-specific agents of the immune system and are especially known for their role in response to bacterial and viral infections [16]. For example, the antiviral activity of bovine BAC5 has been previously reported in the literature [15]. Elevated expression of antimicrobial peptides including cathelicidins has also been found in MSC of cows during mastitis [16, 51], while constitutive expression of *BAC5* and *BAC7.5* in MSC of uninfected goats has also been detected [24]. Moreover, Zhang et al. [52] found similar expressions of *BAC5*, *BAC7.5*, *MAP28* and *MAP34* genes in the mammary glands of healthy and mastitic goats. The inhibitory role of human (LL-37) and swine (protegrin-1) cathelicidins in HIV-1 infection (in vitro study) has also been reported [53, 54]. Furthermore, together with defensins, cathelicidins not only have a broad-spectrum of antimicrobial potency but also play regulatory and effector roles in the innate immune system. Some activate complement or mast cells, induce monocytes, neutrophils, T cells, mast, and immature dendritic cell chemotaxis, as well as cell proliferation, angiogenesis, and many others processes [55]. Information about the expression of cathelicidin genes in lentivirus infection in goats is still limited, thus it is not possible to compare our results with others. As we showed, there were no differences between the tissues of uninfected goats in cathelicidin gene expression. However, we revealed a similar pattern of expression of one cathelicidin—MAP28—at both the mRNA and protein levels, with lower expression in BL/blood serum and MSC/milk of infected animals. Moreover, the concentration of MAP28 was reduced, while that of MAP34 was elevated, but only in the milk of SRLV-infected animals. These two cathelicidins may play vital but contradictory roles in virus infection in the milk of goats. Further study is needed to clarify these roles.

In some cases, we obtained opposite results between mRNA and protein concentrations in both BL/blood serum and MSC/milk. Thus, the discrepancy was found between gene expression at the mRNA level and protein level. In other studies, for example in a study on cytokine expression in HIV infection, increased *IL*-*1β* mRNA expression was found but production of the protein was lacking in astrocytes [56]. Moreover, in our earlier studies [9, 24], we found expression of *IFN*-*γ* and *TNF*-*α* at the mRNA level in BL, with no expression of these proteins in the blood of both SRLV-infected and uninfected goats. The differences in expression patterns at the mRNA and protein levels could be explained by some post-transcriptional or post-translational modifications. To our knowledge, no other studies on this topic have been conducted in small ruminants. Thus, first of all, the microRNAs (miRNAs) responsible for regulating the maturation of APP and cathelicidin mRNAs during SRLV inflammation should be identified, because some of the epigenetic modifications are regulated by miRNAs through degradation or translational silencing of mRNA [57]. This could mean that lentivirus infection influences miRNA gene expression. We can only speculate whether SRLV inhibits this translational process, using mechanisms such as downregulating the expression of receptors, transcription factors, signaling enzymes, or other proteins involved in the regulation of transcription.

In summary, except for a similar study conducted on goats by Czopowicz et al. [7], it is difficult to find any comparative data for APP and cathelicidin expression profiles in small ruminants as a response to SRLV infections. Comparison of gene expression levels in lentivirus infection between species is difficult, especially since in different animal species, different proteins are classified as APP (e.g., TTR, as a negative APP, is an inflammatory marker in humans (where it is routinely measured) and swine, but in goats, TTR’s role has yet to be elucidated) [1, 13, 58]. We only found an in vitro study on APP expressions in goat synovial membrane (GSM) cells isolated from the carpal synovial membrane of colostrum-deprived newborn goat kids [11]. The authors stated that AGP was constitutively produced by GSM cells. They also showed no differences in the AGP gene expression profile between healthy and SRLV-infected 2- to 4-year-old goats with clinical signs of carpal joint disease. Their results are partly in accordance with ours—we found an elevated transcript level of AGP only in MSC, not in BL.

Lower concentrations of SAA, Cp, and MAP28 in milk than in blood indicate the differences in expression profile for APP genes in various biological materials.

The elevated SAA expression in blood serum at the protein level was accompanied by decreased concentrations of SAA and Cp in the milk of infected goats. This finding may suggest that the immune response of the mammary gland differs from the humoral response. The lack of differences between infected and uninfected animals in the concentrations of other APP and cathelicidins in milk and blood may also mean that SRLV can evade the immune system. Moreover, since SAA probably inhibits the production of antibodies and inflammatory reactions, as well as stimulating the differentiation of monocytes to macrophages (which is essential for viral multiplication), elevated expression of this protein could help the virus to continuously infect new cells. The higher concentration of SAA and the unchanged concentration of other APP in the blood serum plus the simultaneous, reduced concentrations of SAA and Cp in the milk of SRLV-infected goats may serve as additive indicators of this infection.

The differences in expression patterns of mRNA and protein levels could be explained by post-transcriptional modifications influenced by the virus, but an epigenetic study is needed to identify these phenomena.

## Abbreviations

AGP: α1-acid glycoprotein
AMP: antimicrobial peptide
APP: acute phase protein
BAC5: bactenecin 5
BAC7.5: bactenecin 7.5
BL: blood leukocyte
CAEV: caprine arthritis encephalitis virus
Cp: ceruloplasmin
CRP: C-reactive protein
ELISA: enzyme-linked immunosorbent assay
Fb: fibrinogen
FIV: feline immunodeficiency virus
GSM: goat synovial membrane
HIV: human immunodeficiency virus
Hp: haptoglobin
IFN-γ: interferon alpha
INRA: Institut National de la Recherche Agronomique
LALBA: alpha-lactalbumin
Lf: lactoferrin
LL-37: human cathelicidin
LSMEANS: Least-squares means
MAP28: cathelicidin 6
MAP34: cathelicidin 7
MSC: milk somatic cell
PBS: phosphate-buffered saline
PFI: Polish Fawn Improved
PPIA: cyclophilin A
PWI: Polish White Improved
SAA: serum amyloid A
SE: standard error
SIV: simian immunodeficiency virus
SRLV: small ruminant lentivirus
TNF-α: tumor necrosis factor alpha
Tf: transferrin
TTR: transthyretin
VMV: visna-maedi virus
